# Echoes through time: amazing inferences from a fossil bat

**DOI:** 10.1186/s40850-024-00193-0

**Published:** 2024-02-04

**Authors:** Lucas J. S. Greville, Lily Hou, Harry A. W. Kumbhani, Beatriz Nogueira e Figueira, Karen J. Vanderwolf, Ryan A. C. Leys, Mathumy Sivatheesan, Thomas P. Pianta, Liam P. McGuire

**Affiliations:** Department of Biology, University of Waterloo, 200 University Avenue, N2L 3G1 Waterloo, ON Canada

## Abstract

Sister to the Chiroptera crown-clade, the 50 million year old *Vielasia sigei* is suggested to have used laryngeal echolocation based on morphometric analyses. We discuss how *Vielasia*’s discovery influences our understanding of the evolution of echolocation in bats and the insights fossils provide to the lives of extinct species.

Laryngeal echolocation is a remarkable adaptation central to the lives of > 1,000 bat species for navigation, finding and capturing prey, communication, and more [1]. Echolocation opens up foraging niches and provides access to resources that otherwise would not be available. Laryngeal echolocation, produced by the bony connections in the larynx, is present in 20 of the 21 families of bats. The exception is the family Pteropodidae which consists of Old World fruit and nectar bats including flying foxes. The evolution of this phylogenetic arrangement was easy enough to square away when bats were organized into two suborders– Megachiroptera (family Pteropodidae) that do not use laryngeal echolocation and Microchiroptera (all other bats) that do. But there is now ample and compelling evidence that bats should be organized into suborder Yinpterochiroptera (family Pteropodidae and six laryngeal echolocating families) and suborder Yangochiroptera (all other laryngeal echolocating families) [2]. This raises the question: was there a single evolution of laryngeal echolocation and subsequent loss in Pteropodidae, or did echolocation evolve independently in the Yinpterochiroptera and Yangochiroptera [3]? Clues to answer this question may lie in the fossil record.

Detailed morphometric measurements of the inner ear have been used to infer the ability to echolocate [4 and references therein]. However, the bat fossil record is quite sparse, and intact fossil material is rare with some species described from a single bone or tooth [5]. Even more challenging, skull material, when available, is typically crushed, limiting inferences that can be made from inner ear morphology [5]. But sometimes a special opportunity is unearthed. In a recent C*urrent Biology* publication, Suzanne Hand and colleagues [4] describe a novel bat species based on fossil remains uncovered in cave sediments of southwestern France. This new bat, *Vielasia sigei*, dates back to the late early Eocene epoch and represents one of the oldest bat fossils at ~ 50 million years old. *Vielasia* is placed sister to, and thus just outside, the lineage of modern bats, sharing a more recent common ancestor with modern bats than any other fossil species. This phylogenetic position allows researchers to study traits that are either shared or differ between *Vielasia* fossils and extant bats to form hypotheses about chiropteran evolution.

The exceptional holotype specimen of *Vielasia* is one of the most complete craniodental records for fossilized bats at over 53% complete (per experimental character matrix) [4]. Importantly, the skull is not crushed as many fossil skulls are. The intact skull allowed researchers to make detailed measurements of critical auditory structures− including the cochlea, basilar membrane, and semicircular canals − providing insight into the hearing capabilities of the species. Comparing these structures to extant bats and other mammals, Hand et al. [4] found that *Vielasia* consistently grouped with laryngeally echolocating species. Amazingly, the research team was able to further infer that *Vielasia* emitted echolocation calls orally (as opposed to some extant species that are nasal emitters), and used low duty cycle, multi-harmonic calls in the range of ~ 30–56 kHz. The authors concluded “there is little to suggest that *Vielasia* used a type of echolocation different from that used by modern [laryngeally-echolocating] bats” [4(p33)] (Fig. 1).

**Fig. 1:**
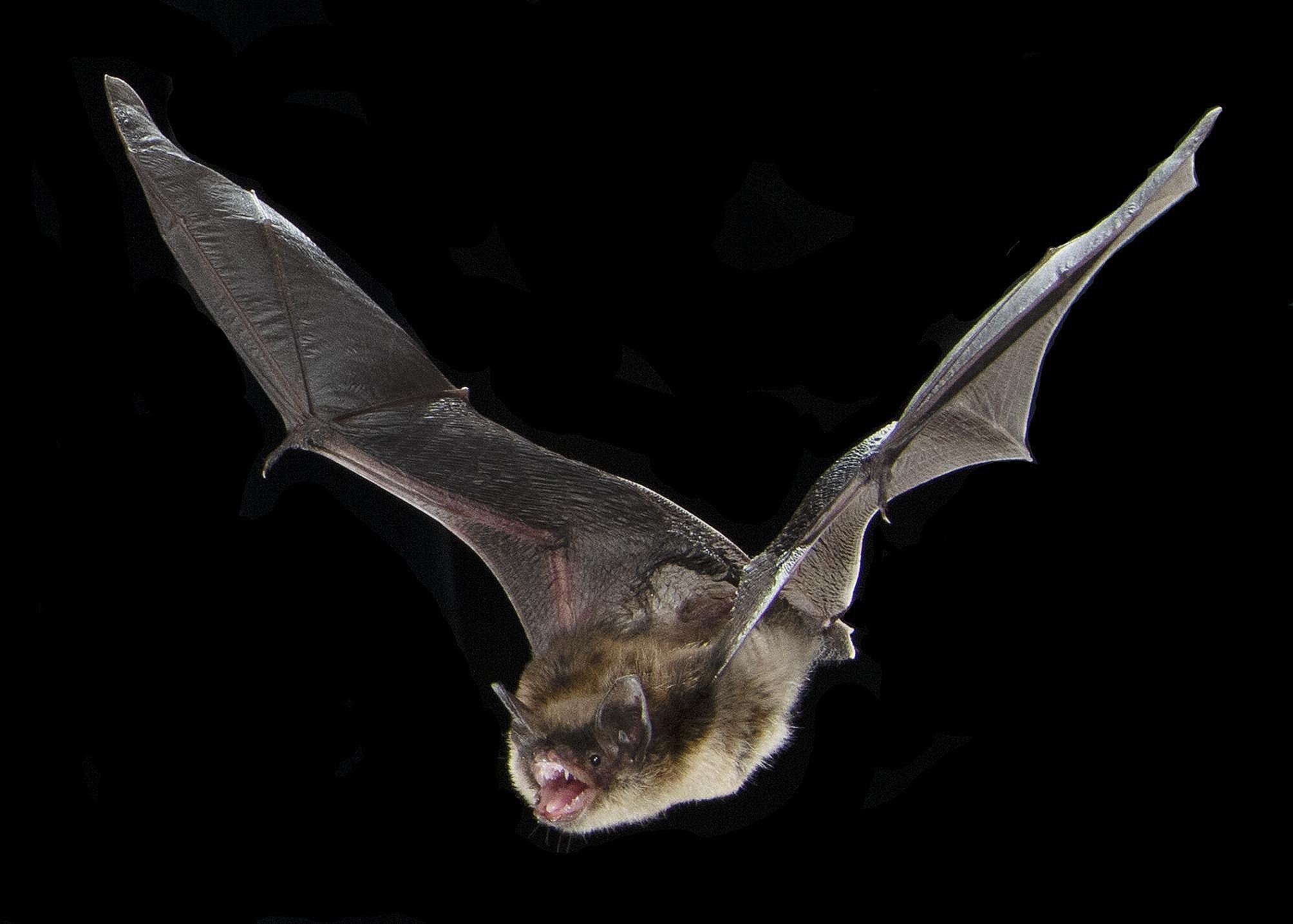
*Myotis lucifugus* orally emitting laryngeal echolocation calls while flying as *Vielasia sigei* is believed to have done. Photo credit Sherri and Brock Fenton

Similar to stratigraphically older and possibly more basal bats, [6] morphometric evidence strongly indicates that *Vielasia* (sister taxon to the crown-clade) was likely capable of laryngeal echolocation [4]. This finding adds to a growing body of literature supporting the ancestral bat as a laryngeal echolocator. There remain multiple scenarios for the evolution of echolocation, either “that advanced echolocation evolved once in the common ancestor of extant bats…but was lost in pteropodids, or that advanced echolocation evolved independently several times in bats, at least once in non-crown bats and twice in extant bat lineages.” [4(p33)] Ultimately, laryngeal echolocation may best be considered as a suite of traits, some of which may have preceded others over evolutionary time [7]. 

Aside from compelling evidence for the evolution of echolocation, the specimens of *Vielasia* allowed Hand et al. [4] to provide many details on the natural history, ecology, and behaviour of the species. For example, *Vielasia* had small eyes that would be unlikely to permit visual hunting at night (further supporting its reliance on echolocation). (see [6], [7]) Long bone analyses suggest the species had an adult body mass of ~ 19 g and mandible morphology indicating the species was likely insectivorous, an estimate remarkably similar to previous predictions of the common ancestor of extant bats [8]. Aside from morphometrics, exceptional opportunity lies in the fact that the remains of at least 23 individuals were discovered together in cave sediment including at least one juvenile. This suggests that at least some bat species had become cave dwelling by the late early Eocene epoch. It is also reasonable to suggest that *Vielasia* may have been a social, group living species that raised their young within the colony. This sociality mirrors that observed in many extant bats [9] and suggests the social nature of Chiroptera may have evolved early in the bat fossil record.

*Vielasia*’s discovery has provided critical insights into the evolution of modern bats. Yet one can’t help but wonder where the discovery of new fossils will lead us. With strong evidence now suggesting that laryngeal echolocation may have evolved only once in Chiroptera, we become forced to reconcile with data that suggest the contrary [e.g., 10]. Presumably echolocation evolved in a transitional manner, but when and how punctuated was that process? Was it the evolution of laryngeal echolocation that gave rise to adaptive radiation in bats? While it is inferred that *Vielasia* was a social species, we can’t know for certain that the individuals interacted with each other or whether they merely died in a common location. Is it even possible to distinguish sociality from common resource use based on the fossil record? The answers to these questions may remain buried in fossils yet discovered. Donald Griffin, the discoverer of echolocation, referred to echolocation as a ‘magic well’ because every time one goes back to the well there is something new to discover. In the same regard, the fossil record can be considered a magic well and it is exciting to think about revelations still to come.

## Conclusions

In an age of technological advancements leading to more detailed and focused biological research, the fossil record continues to be a critical resource for scientists to study evolutionary questions. Morphometric and genetic analyses of fossils can provide us with a detailed view of the lives extinct species once lived.

## Data Availability

No datasets were generated or analysed during the current study.

## Declarations

### Authors’ information

This commentary was a collective effort by trainees (postdoctoral fellows, graduate students, and undergraduates students) in the lab of Dr. Liam McGuire at the University of Waterloo. The lab’s research focuses on the ecology and eco-physiology of temperate bats and birds.
